# On the stability of equilibria in degenerate periodic Hamiltonian systems

**DOI:** 10.1371/journal.pone.0359575

**Published:** 2026-09-29

**Authors:** Nina Xue

**Affiliations:** School of Mathematics and Statistics, Weifang University, Weifang, Shandong, China; Commonwealth Scientific and Industrial Research Organisation, AUSTRALIA

## Abstract

This paper addresses the problem of Lyapunov stability for equilibrium solutions in periodic Hamiltonian systems under degenerate conditions. The degeneracy occurs in resonant cases when the leading coefficient function in the normal form possesses only zeros of high multiplicity, rendering standard criteria inapplicable. By employing Lie normal form theory, we establish two general stability criteria that resolve the stability problem for nearly all such degenerate scenarios. Our results explicitly demonstrate the decisive role of higher-order terms and provide easily verifiable conditions that generalize several known results in the literature.

## Introduction

This work focuses on the dynamics near the origin for a periodically forced Hamiltonian system with one degree of freedom. The Hamiltonian *H*(*x*,*y*,*t*) is taken to be real analytic in the spatial variables (*x*,*y*) near (0,0), continuous and 2π-periodic in *t*, and satisfies the equilibrium conditions $H(0,0,t)=H_{x}(0,0,t)=H_{y}(0,0,t)=0$. I*t*s local Taylor expansion is


$$\begin{array}{c} H(x,y,t)=\sum_{j=2}^{\infty }H_{j}(x,y,t), \end{array}$$ (1)

where


$$\begin{array}{c} H_{j}(x,y,t)=\sum_{\mu +\nu =j}h_{\mu \nu }(t)x^{\mu }y^{\nu },\;j=2,3,\cdots . \end{array}$$

The coefficients $h_{\mu \nu }(t)$ are all 2π-periodic. The corresponding equations of motion are


$$\begin{array}{c} \dot{x}=\frac{\partial H}{\partial y}(x,y,t),\;\dot{y}=-\frac{\partial H}{\partial x}(x,y,t), \end{array}$$ (2)

with the dot indicating the time derivative.

Over the past two decades, the study of the stability of equilibrium solutions in Hamiltonian systems has garnered widespread interest ([1–4]). Understanding the stability of equilibrium positions in Hamiltonian systems is crucial for characterizing their qualitative dynamics. Despite its importance, determining Lyapunov stability for periodic Hamiltonian systems such as system (2) remains an unresolved challenge in many cases. Analytical methods for determining stability are limited to specific circumstances. First, if the linearization has a characteristic exponent with a non-zero real part, the equilibrium is unstable. This leaves the case where the linearized system is stable, with purely imaginary characteristic exponents ±iω,(ω≠0), as the subject of inquiry. Here, the nonlinear part of the system becomes crucial for resolving the stability question.

For the general elliptic case, (that is, the classical situation in which the characteristic exponents are purely imaginary and the Hamiltonian, after suitable normalization, depends on the action variable in a non-degenerate manner, i.e., with a non-vanishing derivative with respect to the action at the origin), seminal results on stability under certain nondegeneracy conditions were established by Arnold ([5]) and Moser ([6]). Their work has spawned a substantial body of subsequent literature on the stability of the trivial solution. Meanwhile, for time-periodic Lagrangian equations, Ortega ([7–10]) developed the third-order approximation method, a technique later extended by other researchers to establish stability for systems like the forced pendulum and singular equations, (by “singular equations” we mean second-order Newtonian periodic differential equations $x^{\prime \prime }+g(x)=f(t)$, where the nonlinear restoring termw *g*(*x*) exhibits a blow-up singularity at some finite spatial value of *x*), one can refer to [11–13] and [14]. A common limitation of these cited results, however, is their inapplicability to the de*g*enerate case central to this paper.

By employing a linear 2π-periodic symplectic transformation, the quadratic Hamiltonian *H*_2_ can be cast in the form


$$\begin{array}{c} H_{2}(x,y)=\frac{\omega }{2}(x^{2}+y^{2}). \end{array}$$

Symplectic action-angle variables are introduced through the canonical transformation defined by


$$\begin{array}{c} x=\sqrt{2r}\cos\varphi ,\;y=\sqrt{2r}\sin\varphi . \end{array}$$

Expressed in *r* and φ, the Hamiltonian (1) takes the form


$$\begin{array}{c} H(r,\varphi ,t)=H_{2}(r)+H_{3}(r,\varphi ,t)+\cdots +H_{j}(r,\varphi ,t)+\cdots , \end{array}$$ (3)

where, by a slight notational abuse, we continue to denote the transformed Hamiltonian by *H*, and


$$\begin{array}{c} H_{2}(r)=\omega r, \end{array}$$

$$\begin{array}{c} H_{j}(r,\varphi ,t)=\sum_{\mu +\nu =j}h_{\mu \nu }(t)(2r{)}^{\frac{j}{2}}\cos^{\mu }\varphi \sin^{\nu }\varphi ,\;j\geq 3. \end{array}$$

By applying the Lie normal form process, a formal, symplectic, and 2π-periodic change of variables brings (3) into the following form

(1) In the case of an irrational frequency ω, the normalized Hamiltonian reduces to $H(r)=\omega r+A_{4}r^{2}+\cdots +A_{2n}r^{n}+\cdots$, indicating that it depends solely on the action variable *r*;
(2) Conversely, in the resonant case where ω is rational, i.e., ω=p/k for coprime integers *p*, *k* > 0 (corresponding to a resonance of order *k*), the Hamiltonian takes the form

$$\begin{array}{c} H=H(r,\varphi )=A_{4}r^{2}+\cdots +A_{2l}r^{l}+H_{k}(r,k\varphi )+\cdots +H_{m}(r,k\varphi )+\cdots , \end{array}$$

where 2*l* is the largest even integer smaller than *k*, and m≥k is the order of normalization. The formal statement and proof are given in Lemma 1.

Since stability analysis typically requires the normal form only up to a finite order, the formal transformation becomes convergent in this context. Then, by the Moser twist theorem (see [6]), the existence of any non-vanishing coefficient $A_{2j}\neq 0$ (for some j≥2) implies the Lyapunov stability of the origin in system (2). Although it has been shown [3] that the Hamiltonian (3) can be analytically linearized to $H_{2}=\omega r$ ensuring stability when ω is Diophantine (a number ω is called Diophantine if there exist constants γ>0 and τ≥1 such that $|q\omega -p|\geq \gamma |q{|}^{-\tau }$ for all integers *p*, *q* with q≠0). and all nonlinear coefficients *A*_2*j*_ vanish, this conclusion does not extend to non-Diophantine irrational frequencies, whose stability remains unresolved.

There exists an extensive literature addressing the stability problem in the resonant case where ω is rational (see [1,2,15–20]). A typical approach begins with the derivation of the Lie normal form to a certain order. This is followed by the application of either Chetaev’s/Lyapunov’s theorems to demonstrate instability, or the Moser twist theorem to verify stability. Within this framework, Cabral and Meyer [16] established a general stability criterion. This result can be stated as follows

**Theorem A**  *Consider the Hamiltonian system defined by*


$$\begin{array}{c} \dot{r}=\frac{\partial K}{\partial \varphi }(r,\varphi ,t),\;\dot{\varphi }=-\frac{\partial K}{\partial r}(r,\varphi ,t) \end{array}$$

*with the Hamiltonian* $K(r,\varphi ,t)=\psi (\varphi )r^{n}+O(r^{n+\frac{1}{2}})$ *as* $r\to 0^{+}$*, here* $n=\frac{m}{2}$ *with* m≥3 *be an integer. Assume K is analytic in* $\sqrt{r},\varphi ,t$*, and is* τ*-periodic in* φ *and T-periodic in t. Under these conditions, the equilibrium r = 0 is stable if* ψ(φ)≠0 *for all* φ∈ℝ*, and unstable if* ψ(φ) *possesses a simple zero.*

Our analysis also concerns the resonant case, namely when $\omega =\frac{p}{k}$ is rational. We restrict our attention to the degenerate situation where $A_{4}=\cdots =A_{2l}=0$, since otherwise stability is immediate. In this setting, the Hamiltonian (3) transforms into the structure described in Theorem A. For an odd-order resonance, *k* = 2*n* + 1, the Hamiltonian (3) reduces to


$$\begin{array}{c} H=Br^{\frac{2n+1}{2}}\cos(2n+1)\varphi +\tilde{H}(r,\varphi ,t), \end{array}$$

this Hamiltonian is analytic, and $\tilde{H}=O(r^{n+1})$ is periodic in *t*. Theorem A thus guarantees Lyapunov instabili*t*y for B≠0. Having addressed the odd-order case, we turn to the situation where *B* = 0 or the resonance order is even (*k* = 2*n* + 2). Here, the Hamiltonian (3) takes the form


$$\begin{array}{c} H=r^{n+1}\left(C+D\cos2(n+1)\varphi \right)+\tilde{H}(r,\varphi ,t). \end{array}$$

The analytic Hamiltonian features a remainder $\tilde{H}=O(r^{n+\frac{3}{2}})$ that is periodic in *t*. Under the condition |*C*| > |*D*|, Theorem A guarantees *t*he Lyapunov stability of the origin. Conversely, instability occurs when |*C*| < |*D*|. However, Theorem A offers no conclusion when |*C*| = |*D*|, as all zeros of the function C+Dcos2(n+1)φ are of multiplicity two. This is the precise case that falls outside the scope of the standard theory and will be investigated in this work.

We further extend our analysis to the general degenerate case, where all zeros of ψ(φ) in Theorem A possess multiplicities greater than one. This scenario, in which Theorem A is no longer applicable, is commonly referred to in the literature as the degenerate case. Hence, to resolve the stability problem in the degenerate case, it becomes essential to incorporate terms in the Hamiltonian K(r,φ,t) of order higher than $r^{n}$.

Several results exist for stability analysis in degenerate settings. To our knowledge, the pioneering work is due to Markeyev [18,19], who derived sufficient conditions for both stability and instability in the case of fourth-order resonance. His analysis applied to both periodic one-degree-of-freedom and autonomous two-degree-of-freedom Hamiltonian systems, employing the Moser twist theorem and Lyapunov’s method, respectively. Following this line of research, Mansilla and Vidal [17] further examined even-order resonances, and Bardin [15] recently developed general criteria for degeneracy using Lie normal forms. The core method unifying these results is the derivation of stability conditions from a normal form truncated at an appropriate order.

For the class of degenerate periodic Hamiltonian systems with one degree of freedom in which the normalized action-dependent part vanishes identically and the leading angular coefficient possesses only zeros of finite multiplicity, Theorems 1 and 2 in the Main results section provide a complete stability classification except for the measure-zero set of parameters where $\varphi_{0}$ and $\varphi_{1}$ share common zeros. Moreover, our approach offers distinct advantages over existing ones. Compared to [17] and [18], we provide stability criteria applicable to resonances of arbitrary order. Furthermore, unlike the methodology in [15], our resulting criteria are straightforward to verify and circumvent the need for computing the intricate main part required in their analysis.

To substantiate the claimed novelty, we present the following formal comparison.

**Proposition 1.** *Whenever the hypotheses of Bardin (2015) or Mansilla and Vidal (2013) are satisfied, the hypotheses of our Theorems 1 and 2 are also satisfied. The converse is not true.*

The proof of this proposition follows by observing that the conditions in [15] and [17] require a non-degenerate twist at the lowest non-vanishing order, whereas our criteria allow for zeros of arbitrary finite multiplicity. A representative example is given in Example below, which falls outside the scope of Bardin (2015) and Mansilla and Vidal (2013) but is covered by our Theorem 1.

The principal difficulty encountered in this work is the complete vanishing of the action-dependent part of the normalized Hamiltonian, which prevents the direct application of standard twist theorems. To overcome this, we developed a two-step normalization procedure combined with a carefully chosen canonical transformation that restores a twist-like structure at a higher order. The difficulties related to handling zeros of arbitrary (even or odd) multiplicity and the need for distinct instability proofs are addressed by analyzing the sign of the higher-order angular coefficient $\psi_{1}(\varphi )$ near each zero of the leading coefficient $\psi_{0}(\varphi )$.

The remainder of this paper is structured as follows. The Normal forms and definitions section reviews prerequisite concepts regarding resonances and normal forms. Our main results Theorems 1 and 2 along with pertinent remarks, are presented in the Main results section. Their proofs are detailed in the Proofs of Theorem 1 and Theorem 2 section. The Examples section provides several illustrative examples demonstrating how our theorems generalize existing results. Finally, The Conclusion section concludes the paper with a summary of contributions and future directions.

## Normal forms and definitions

The linearized system of (2) is assumed stable, possessing purely imaginary characteristic exponents ±iω for some ω∈ℝ. Let


$$\begin{array}{c} H^{m}(r,\varphi ,t)=H_{2}(r)+H_{3}(r,\varphi ,t)+\cdots +H_{m}(r,\varphi ,t). \end{array}$$ (4)

With $H_{j}\;(j=2,\cdots ,m)$ are given by (), and given the essential role of resonance in stability questions, it is essential to provide a precise definition.

**Definition 1.** *System (2) is said to exhibit a resonance of order k if there exists a minimal positive integer k such that* kω∈ℤ*. Conversely, if* kω∉ℤ *for all integers k with* 1≤|k|≤s*, the system is said to be non-resonant up to order s.*

Consider the set


$$\begin{array}{c} M_{\omega }=\{k\in \mathbb{Z}:k\omega \in \mathbb{Z}\} \end{array}$$

associated with the frequency ω. The condition $M_{\omega }=\{0\}$ is equivalent to the linear independence of ω and 1 over ℚ, meaning system (2) is non-resonant. Conversely, if $M_{\omega }\neq \{0\}$, the system is resonant. In this case, the resonance order is defined as the minimal positive integer *k* in $M_{\omega }$. We then have the following lemma.

**Definition 2.** *A Hamiltonian* $H^{m}$ *is in Lie normal form if it commutes with the quadratic part H*^*2*^ *modulo the time variable, i.e., it satisfies the condition*


$$\begin{array}{c} \{H_{2},H^{m}\}-\frac{\partial H^{m}}{\partial t}=0, \end{array}$$ (5)

where {,} denotes the Poisson bracket [21].

**Lemma 1.** *Let* $H^{m}$ *(m is a fixed integer greater than 2) be in Lie normal form, then*

(i) *for* $M_{\omega }=\{0\}$, $H^{m}$ *reduces to a function* $H^{m}(r)$ *of the action alone;*
(ii) *for* $M_{\omega }=k\mathbb{Z}\neq \{0\}$, $H^{m}$ *admits the structure*

$$\begin{array}{c} H^{m}=H^{m}(r,\varphi )=H_{4}(r)+\cdots +H_{2l}(r)+H_{k}(r,k\varphi )+\cdots +H_{m}(r,k\varphi ), \end{array}$$

*with 2l < k the preceding maximal even integer and* m≥k.

From Definition 2 for $H_{2}=\omega r$, Eq (5) reduces to


$$\begin{array}{c} \omega \frac{\partial H^{m}}{\partial \varphi }=\frac{\partial H^{m}}{\partial t}. \end{array}$$ (6)

Assume that


$$\begin{array}{c} H^{m}(r,\varphi ,t)=\sum_{j=2}^{m}\sum_{\mu +\nu =j}h_{\mu \nu }(t)e^{i(\mu -\nu )\varphi }r^{\frac{j}{2}}, \end{array}$$

then Eq (6) gives us


$$\begin{array}{c} \sum_{j=2}^{m}\sum_{\mu +\nu =j}i\omega (\mu -\nu )h_{\mu \nu }(t)e^{i(\mu -\nu )\varphi }r^{\frac{j}{2}}=\sum_{j=2}^{m}\sum_{\mu +\nu =j}h_{\mu \nu }^{\prime }(t)e^{i(\mu -\nu )\varphi }r^{\frac{j}{2}}. \end{array}$$

This implies that each periodic coefficient $h_{\mu \nu }(t)$ satisfies


$$\begin{array}{c} h_{\mu \nu }^{\prime }(t)=i\omega (\mu -\nu )h_{\mu \nu }(t), \end{array}$$

and hence admits the solution


$$\begin{array}{c} h_{\mu \nu }(t)=C_{\mu \nu }e^{i\omega (\mu -\nu )t} \end{array}$$

for some constant $C_{\mu \nu }\in \mathbb{C}.$

This representation allows us to rewrite the Hamiltonian as


$$\begin{array}{c} H^{m}(r,\varphi ,t)=\sum_{j=2}^{m}\sum_{\mu +\nu =j}C_{\mu \nu }e^{i(\mu -\nu )(\varphi +\omega t)}r^{\frac{j}{2}}. \end{array}$$

The reality of $H^{m}$ implies $C_{\mu \nu }={\overset{―}{C}}_{\nu \mu }$. Furthermore, its 2π-periodicity in *t* restricts non-zero terms to those satisfying (μ−ν)ω∈ℤ, or equivalently, $\mu -\nu \in M_{\omega }$. Consequently, for $M_{\omega }=\{0\}$,we have μ=ν, so $H^{m}=H^{m}(r)$; for $M_{\omega }=k\mathbb{Z}\neq \{0\}$, we have μ−ν∈kℤ, so $H^{m}=H^{m}(r,k(\varphi +\omega t)).$

For the resonant case $M_{\omega }=k\mathbb{Z}\neq \{0\}$, we introduce a canonical transformation defined by


$$\begin{array}{c} \tilde{r}=r,\;\tilde{\varphi }=\varphi +\omega t. \end{array}$$

Explicitly, the transformation is generated by


$$\begin{array}{c} S(\tilde{r},\varphi ,t)=\tilde{r}\cdot (\varphi +\omega t), \end{array}$$

i.e.,


$$\begin{array}{c} r=\frac{\partial S}{\partial \varphi },\;\tilde{\varphi }=\frac{\partial S}{\partial \tilde{r}}. \end{array}$$

The relation $\frac{\partial S}{\partial t}=\tilde{r}\cdot \omega$ yields the new Hamiltonian


$$\begin{array}{c} \tilde{H}(\tilde{r},\tilde{\varphi })=\sum_{j=2}^{l}{\tilde{H}}_{2j}(\tilde{r})+{\tilde{H}}_{k}(\tilde{r},k\tilde{\varphi })+\cdots +{\tilde{H}}_{m}(\tilde{r},k\tilde{\varphi }). \end{array}$$

This transformation is introduced to eliminate the explicit time dependence from the angular variable at the leading order, thereby simplifying the application of the Moser twist theorem. The new Hamiltonian becomes autonomous in $\tilde{\varphi }$ at the leading order because the time-dependent terms have been absorbed into the new angle variable. Although some higher-order terms may still depend on the new angle, the leading-order terms that govern the twist condition are independent of it, which is sufficient for the subsequent stability analysis.

## Main results

### Overview of the strategy

Before stating the main theorems, we outline the four main steps of our approach:

1. Derive the Birkhoff–Gustavson normal form and introduce action-angle variables.
2. Identify the degeneracy in the action-dependent part (i.e., the vanishing of all coefficients *A*_2*j*_).
3. Apply a canonical transformation that removes the leading-order time dependence from the angle, restoring an autonomous structure at the dominant order.
4. Apply either the Moser twist theorem (for stability) or Chetaev’s theorem (for instability) depending on the parity of the multiplicity of zeros of $\psi_{0}(\varphi )$.

### Stability criteria

We begin by establishing two general stability criteria applicable to Hamiltonian of the form


$$\begin{array}{c} H(r,\varphi ,t)=r^{\alpha }\psi_{0}(\varphi )+r^{\alpha +\gamma }\psi_{1}(\varphi )+O(r^{\alpha +\gamma +\frac{1}{2}}), \end{array}$$ (7)

where $\alpha =\frac{m}{2},\;m\geq 3;\gamma =\frac{n}{2},\;n\geq 1.$

This paper focuses on the stability problem for the origin of the Hamiltonian (7) under degenerate conditions, specifically when the leading coefficient function $\psi_{0}(\varphi )$ possesses only zeros of multiplicity greater than one.

We first present a criterion for the case where all zeros of $\psi_{0}(\varphi )$ are of even multiplicity.

**Theorem 1.** *Consider the Hamiltonian system*


$$\begin{array}{c} \dot{\varphi }=\frac{\partial H}{\partial r}(r,\varphi ,t),\;\dot{r}=-\frac{\partial H}{\partial \varphi }(r,\varphi ,t) \end{array}$$ (8)

*where the Hamiltonian function is given by (7). Assume that all zeros of the function* $\psi_{0}(\varphi )$ *have even multiplicity. Then the following stability properties hold for the equilibrium r = 0.*

*(A) (Stability Criterion) If, for every zero* $\varphi_{0}$ *of* $\psi_{0}(\varphi )$ *with multiplicity m > 1, the condition*


$$\begin{array}{c} \psi_{0}^{\left(m\right)}(\varphi_{0})\psi_{1}(\varphi_{0})>0, \end{array}$$

*is satisfied, then the equilibrium r = 0 is Lyapunov stable.*

*(B) (Instability Criterion) If* $\psi_{1}(\varphi )\neq 0$ *at every zero of* $\psi_{0}(\varphi )$*, and there exists at least one zero* $\varphi_{0}$ *of* $\psi_{0}(\varphi )$ *with multiplicity m > 1 such that*


$$\begin{array}{c} \psi_{0}^{\left(m\right)}(\varphi_{0})\;\psi_{1}(\varphi_{0})<0, \end{array}$$

*then the equilibrium r = 0 is Lyapunov unstable.*

We make two observations regarding this theorem. First, in the even-multiplicity case, it is possible to select higher-order terms (beyond $r^{\alpha }$) to achieve either a stable or an unstable configuration. To illustrate this, we examine a system with Hamiltonian


$$\begin{array}{c} H=(1+\sin\varphi )r^{2}+ar^{3}, \end{array}$$

Here, the Hamiltonian has been brought into normal form with a constant coefficient *a*. The leading coefficient function $\psi_{0}(\varphi )=1+\sin\varphi$ clearly has zeros of multiplicity two.

Stability holds for *a* = 1, which follows from Lyapunov’s theorem upon choosing *V* = *H* as a Lyapunov function.

For a=−1, the equilibrium solution is unstable. To see this, examine the zero-energy level set *H* = 0, which consists of the equilibrium line *r* = 0 and the curve r=1+sinφ. On the latter, the equations of motion become


$$\begin{array}{c} \dot{\varphi }=-(1+\sin\varphi {)}^{2},\;\dot{r}=-r^{2}\cos\varphi . \end{array}$$

If we put $\varphi (0)=-\frac{\pi }{2}-\mu ,$ where 0<μ≪1, then $r(0)=2\sin^{2}\frac{\mu }{2}\sim \mu^{2}.$ Within the third quadrant $\left(-\pi <\varphi <-\frac{\pi }{2}\right)$, the angle φ decreases monotonically while *r* increases monotonically from an initially small value *r*(0) toward *r* = 1. This behavior confirms the instability of the equilibrium.

The example illustrates that resolving the stability problem in degenerate settings necessarily requires analyzing the higher-order term $r^{\alpha +\gamma }\psi_{1}(\varphi )$ in the Hamiltonian (7).

Second, Theorem 1 is not directly applicable if $\psi_{0}(\varphi )$ and $\psi_{1}(\varphi )$ have common zeros. This scenario represents an even deeper level of degeneracy, where stability analysis necessitates the consideration of terms beyond $r^{\alpha +\gamma }$ in (7). Nevertheless, the same analytical framework developed here can be extended to address such cases.

We now turn to the complementary case where $\psi_{0}(\varphi )$ has a zero of odd multiplicity greater than one. The corresponding stability result is as follows.

**Theorem 2.** *If there exists some* $\varphi_{0}$ *such that*


$$\begin{array}{c} \psi_{0}(\varphi_{0})=\cdots =\psi_{0}^{\left(m-1\right)}(\varphi_{0})=0,\;\psi_{0}^{\left(m\right)}(\varphi_{0})\neq 0, \end{array}$$

*and*


$$\begin{array}{c} \psi_{0}^{\left(m\right)}(\varphi_{0})\psi_{1}^{\prime }(\varphi_{0})>0, \end{array}$$

*where m > 1 is odd, then the equilibrium r = 0 of the Hamiltonian system (8) is Lyapunov unstable.*

We postpone the proofs of Theorems 1 and 2 to the Proofs of Theorem 1 and Theorem 2 section.

**Remark 1.** *When* $\psi_{0}(\varphi )$ *and* $\psi_{1}(\varphi )$ *share common zeros, the sign of the combined term* $\psi_{0}(\varphi )+r^{\gamma }\psi_{1}(\varphi )$ *near such zeros is not determined by the leading order alone, and our current theorems do not cover this case. This is a codimension-one phenomenon, and a separate analysis (possibly involving higher-order terms or blow-up techniques) would be required. We note that this limitation is common to all existing degenerate-resonance criteria in the literature.*

**Remark 2.** *The conclusions of Theorems 1 and 2 are structurally stable with respect to higher-order terms. Adding sufficiently small higher-order terms in r does not alter the sign of the dominant expression near the zeros of* $\psi_{0}$*, and therefore the conclusions remain valid. This follows from compactness and continuity arguments, since the conditions in the theorems depend only on the signs of* $\psi_{0}^{\left(m\right)}(\varphi_{0})$ *and* $\psi_{1}(\varphi_{0})$ *(or* $\psi_{1}^{\prime }(\varphi_{0})$*), which are preserved under sufficiently small perturbations.*

We now apply the preceding analysis to the original system (2). Assume its linearization has characteristic exponents ±iω satisfying a resonance of order *k*. Introducing action-angle variables and applying the Lie normalization process up to order $r^{\frac{m}{2}}\;(m>k)$, Lemma 1 allows us to express the Hamiltonian as


$$\begin{array}{c} H=A_{4}r^{2}+\cdots +A_{2l}r^{l}+\sum_{j=k}^{m}H_{j}(r,\varphi )+O(r^{\frac{m+1}{2}}), \end{array}$$ (9)

with 2*l* < *k* being the preceding maximal even integer and m≥k. Lemma 1 also implies that each component $H_{j}\;(k\leq j\leq m)$ in (9) takes the form


$$\begin{array}{c} H_{j}=\left(A_{j}+\sum_{s=1}^{\left[\frac{j}{k}\right]}(B_{js}\cos(sk\varphi )+C_{js}\sin(sk\varphi ))\right)r^{\frac{j}{2}},\;k\leq j\leq m, \end{array}$$

where $A_{j},B_{js},C_{js}$ are some real numbers.

In light of the Introduction section, the stability question reduces to the case $A_{4}=\cdots =A_{2l}=0$ in (9), any deviation would imply stability trivially. With this normalization, we begin by examining the case where $H_{k}≢0$. If the resonance order *k* is odd, then $A_{k}=0$, and stability can be determined directly by Theorem A. Therefore, for a non-trivial $H_{k}$, it suffices to consider the case where *k* is even.

Under the assumptions that *k* is even and $H_{k}$ is non-vanishing, it follows that


$$\begin{array}{c} H_{k}=(A_{k}+B_{k1}\cos(k\varphi )+C_{k1}\sin(k\varphi ))r^{\frac{k}{2}}. \end{array}$$

We now focus on the degenerate scenario, defined by $|A_{k}|=\sqrt{B_{k1}^{2}+C_{k1}^{2}}$, we have


$$\begin{array}{c} H_{k}(r,\varphi )=2A_{k}\cos^{2}\frac{k\varphi +\tilde{\varphi }}{2}r^{\frac{k}{2}}:=\psi_{0}(\varphi )r^{\frac{k}{2}}. \end{array}$$

Clearly, all zeros of $\psi_{0}(\varphi )=2A_{k}\cos^{2}\frac{k\varphi +\tilde{\varphi }}{2}$ have multiplicity two, by including terms of order higher than $r^{\frac{k}{2}}$, Theorem 1 becomes applicable. Consequently, the stability problem for the origin of (2) in this degenerate case is therefore fully resolved.

We next address the case $H_{k}\equiv 0$, which directly leads to the analysis of Hamiltonian (7). To demonstrate that the conditions of Theorem 1 (even *m* > 2) and Theorem 2 (odd *m* > 1) are both non-vacuous and realizable, we present two concrete examples.

First, suppose $H_{j}\equiv 0$ for k≤j≤3k−1, but $H_{3k}≢0$ with (3k≤m). Then *H*_3*k*_ is given by


$$\begin{array}{cc} H_{3k}(r,\varphi ) & =(A_{3k}+B_{3k1}\cos k\varphi +C_{3k1}\sin k\varphi +B_{3k2}\cos2k\varphi +C_{3k2}\sin2k\varphi \\ & \;+B_{3k3}\cos3k\varphi +C_{3k3}\cos3k\varphi )r^{\frac{3k}{2}}. \end{array}$$

In particular, if


$$\begin{array}{c} A_{3k}=B_{3k2}=C_{3k1}=C_{3k2}=C_{3k3}=0,\;B_{3k1}-3B_{3k3}=0,\;B_{3k3}\neq 0, \end{array}$$

then


$$\begin{array}{c} H_{3k}(r,\varphi )=4B_{3k3}\cos^{3}(k\varphi )r^{\frac{3k}{2}}:=\psi_{0}(\varphi )r^{\frac{3k}{2}}. \end{array}$$

Clearly, all zeros of


$$\begin{array}{c} \psi_{0}(\varphi )=4B_{3k3}\cos^{3}k\varphi \end{array}$$

have multiplicity three. Hence, this configuration falls under the purview of Theorem 2, where the multiplicity *m* is an odd integer greater than one.

In a similar vein, consider the case where $H_{k}\equiv 0,H_{k+1}\equiv 0,\cdots ,H_{4k-1}\equiv 0$ and $H_{4k}≢0,$ with 4k≤m. This leads to the expression


$$\begin{array}{cc} H_{4k}(r,\varphi ) & =(A_{4k}+B_{4k1}\cos k\varphi +C_{4k1}\sin k\varphi +B_{4k2}\cos2k\varphi +C_{4k2}\sin2k\varphi \\ & \;+B_{4k3}\cos3k\varphi +C_{4k3}\cos3k\varphi +B_{4k4}\cos4k\varphi +C_{4k4}\cos4k\varphi )r^{2k}. \end{array}$$

In particular, if


$$\begin{array}{c} B_{4k1}=B_{4k3}=C_{4k1}=C_{4k2}=C_{4k3}=C_{4k4}=0, \end{array}$$

$$\begin{array}{c} \;A_{4k}-3B_{4k4}=0,\;B_{4k2}-4B_{4k4}=0,\;B_{4k4}\neq 0, \end{array}$$

then


$$\begin{array}{c} H_{4k}(r,\varphi )=8B_{4k4}\cos^{4}(k\varphi )r^{2k}:=\psi_{0}(\varphi )r^{2k}. \end{array}$$

Clearly, the function $\psi_{0}(\varphi )=8B_{4k4}\cos^{4}k\varphi r^{2k}$ has zeros of multiplicity four. Thus, this example belongs to the regime covered by Theorem 1, where the multiplicity *m* is an even integer greater than two.

### Proofs of Theorem 1 and Theorem 2

The proof of Theorem 1 proceeds as follows. Stability is verified via the Moser twist theorem [6], and instability is proved using Chetaev’s theorem [21]. We begin with the following lemma.

**Lemma 2.** *Let* $H_{0}(r,\varphi )=r^{\alpha }\psi_{0}(\varphi )+r^{\alpha +\gamma }\psi_{1}(\varphi )$ *be the truncated Hamiltonian with* $\alpha =\frac{m}{2}$, m≥3 *and* $\gamma =\frac{n}{2}$*,* n≥1*. Assume that the conditions of part* (A) *of Theorem 1 hold, so that H*_*0*_ *> 0 for sufficiently small r > 0. Then there exists an analytic symplectic change of variables* (r,φ)↦(I,θ)*, defined for* $0<H_{0}\ll 1$*, such that:*

(i) *The action variable is given by* $I(h)=\frac{1}{2\pi }\int_{0}^{2\pi }r(\varphi ,h)\;d\varphi$*, where* r(φ,h) *is the unique solution of* $H_{0}(r,\varphi )=h$;
(ii) *The angle variable* θ *satisfies* {I,θ}=1*, so that the transformation is symplectic.*

**Proof.** Under the conditions of (A), *H*_0_ is positive definite for small *r* > 0. For each fixed energy level *h* > 0 sufficiently small, the equation $H_{0}(r,\varphi )=h$ defines a unique closed curve in the (r,φ)-plane. By the implicit function theorem, this curve can be written as $r=r_{0}(\varphi ,h)$. The action variable is defined as the area enclosed by this curve divided by 2π:


$$\begin{array}{c} I(h)=\frac{1}{2\pi }\oint r_{0}(\varphi ,h)\;d\varphi =\frac{1}{2\pi }\int_{0}^{2\pi }r(\varphi ,h)\;d\varphi . \end{array}$$

Since $\frac{\partial H_{0}}{\partial r}>0$ for small *r* > 0, the function h↦I(h) is strictly increasing with *I*(0) = 0 and $\frac{dI}{dh}>0$. By the inverse function theorem, the inverse function *h* = *h*(*I*) exists and is analytic for 0<I≪1.

The angle variable θ is defined by the standard formula


$$\begin{array}{c} \theta =\frac{2\pi }{T(h)}\int_{\varphi_{0}}^{\varphi }\frac{d\varphi }{\frac{\partial H_{0}}{\partial r}(r_{0}(\varphi ,h),\varphi )}, \end{array}$$

where


$$\begin{array}{c} T(h)=\int_{0}^{2\pi }\frac{d\varphi }{\frac{\partial H_{0}}{\partial r}(r_{0}(\varphi ,h),\varphi )} \end{array}$$

is the period of the motion. A direct computation shows that {I,θ}=1, confirming the symplectic nature of the transformation.

We now turn to the proof of Theorem 1.

**Proof of Theorem 1** The stability assertion in (A) is proved using a method analogous to [15,17]. The first step is to analyze the truncated Hamiltonian $H_{0}=r^{\alpha }\psi_{0}(\varphi )+r^{\alpha +\gamma }\psi_{1}(\varphi ).$ Under the conditions of (A), we can assume, without loss of generality, that


$$\begin{array}{c} \psi_{0}^{\left(m\right)}(\varphi_{0})>0,\;\psi_{1}(\varphi_{0})>0, \end{array}$$

with *m* > 1 being the multiplicity of $\varphi_{0}$. A Taylor expansion of $\psi_{0}(\varphi )$ and $\psi_{1}(\varphi )$ at $\varphi_{0}$ gives


$$\begin{array}{cc} H_{0} & =r^{\alpha }\psi_{0}(\varphi )+r^{\alpha +\gamma }\psi_{1}(\varphi )\\ & =r^{\alpha }\frac{\psi_{0}^{\left(m\right)}(\varphi_{0})}{m!}(\varphi -\varphi_{0}{)}^{m}+r^{\alpha +\gamma }\psi_{1}(\varphi_{0})\\ & \;+r^{\alpha }O(\varphi -\varphi_{0}{)}^{m+1}+r^{\alpha +\gamma }O(\varphi -\varphi_{0}),\;|\varphi -\varphi_{0}|\ll 1. \end{array}$$

Consequently, for small *r* > 0, the function *H*_0_ is positive definite. In addition,


$$\begin{array}{c} \frac{d\varphi }{dt}=\frac{\partial H_{0}}{\partial r}=\alpha r^{\alpha -1}\left(\psi_{0}(\varphi )+\frac{\alpha +\gamma }{\alpha }r^{\gamma }\psi_{1}(\varphi )\right)>0 \end{array}$$

for *r* > 0 small enough.

Consider the energy level set $H_{0}(r,\varphi )=h_{0}$. By the implicit function theorem, we obtain a function $r=r_{0}(\varphi ,h_{0})$. For a perturbed energy $h=h_{0}+\mu$ with |μ|≪1, the solution r(φ,h) of $H_{0}(r,\varphi )=h$ can be expanded in powers of μ as follows


$$\begin{array}{c} r(\varphi ,h)=r_{0}(\varphi ,h_{0})+\mu r_{1}(\varphi ,h_{0})+O(\mu^{2}) \end{array}$$

with


$$\begin{array}{c} r_{1}(\varphi ,h_{0})={\left(\frac{\partial H_{0}}{\partial r}\right)}^{-1}>0. \end{array}$$

By Lemma 2, we proceed to introduce the action-angle variables associated with *H*_0_. The action variable, denoted by *I*(*h*) and satisfying *I*(0)=0, is given by


$$\begin{array}{c} I=\frac{1}{2\pi }\int_{0}^{2\pi }r(\varphi ,h)d\varphi =I_{0}+\mu I_{1}+O(\mu^{2}), \end{array}$$

where


$$\begin{array}{c} I_{0}=\frac{1}{2\pi }\int_{0}^{2\pi }r_{0}(\varphi ,h_{0})d\varphi ,\;I_{1}=\frac{1}{2\pi }\int_{0}^{2\pi }r_{1}(\varphi ,h_{0})d\varphi >0. \end{array}$$

The condition $I_{1}\neq 0$ implies, via the inverse function theorem, that the inverse function *h* = *h*(*I*) exists and is analytic for for 0<I≪1.

Expressed in the action-angle coordinates (I,θ), the Hamiltonian becomes


$$\begin{array}{c} H=h(I)+h_{1}(\theta ,I,t), \end{array}$$

with $h_{1}(\theta ,I,t)=o(h(I))$ analytic in its arguments and periodic in θ (period 2π) and *t* (period *T*).

By the Moser twist theorem, stability of *I* = 0 is guaranteed provided the following non-degeneracy condition holds:


$$\begin{array}{c} \frac{d^{2}h}{dI^{2}}\neq 0. \end{array}$$ (10)

Straightforward calculations yield


$$\begin{array}{c} \frac{d^{2}h}{dI^{2}}=\frac{\lambda^{3}}{2\pi }\int_{0}^{2\pi }{\left(\frac{\partial H_{0}}{\partial r}\right)}^{-3}\frac{\partial^{2}H_{0}}{\partial r^{2}}d\varphi , \end{array}$$

where


$$\begin{array}{c} \lambda =\frac{2\pi }{\int_{0}^{2\pi }{\left(\frac{\partial H_{0}}{\partial r}\right)}^{-1}d\varphi }. \end{array}$$

Since $\frac{\partial H_{0}}{\partial r}>0,$ we have λ>0 for sufficiently small *r* > 0. Direct calculation gives


$$\begin{array}{c} \frac{\partial^{2}H_{0}}{\partial r^{2}}=\alpha (\alpha -1)r^{\alpha -2}\left(\psi_{0}(\varphi )+\frac{\left(\alpha +\gamma )(\alpha +\gamma -1\right)}{\alpha (\alpha -1)}r^{\gamma }\psi_{1}(\varphi )\right)>0 \end{array}$$

for sufficiently small *r* > 0. Therefore, condition (10) holds, completing the proof of part (A).

Next, we prove part (B). Let


$$\begin{array}{c} \psi (r,\varphi )=\psi_{0}(\varphi )+r^{\gamma }\psi_{1}(\varphi ), \end{array}$$

then the Hamiltonian (7) reads


$$\begin{array}{c} H(r,\varphi ,t)=r^{\alpha }\psi (r,\varphi )+O(r^{\alpha +\gamma +\frac{1}{2}}), \end{array}$$

where $\alpha =\frac{m}{2},\;m\geq 3;\gamma =\frac{n}{2},\;n\geq 1.$

Under the hypotheses of (B), for any fixed sufficiently small *r* > 0, all real roots of the equation $\psi (r,\varphi )=\psi_{0}(\varphi )+r^{\gamma }\psi_{1}(\varphi )=0$ are simple. To verify this, take any zero $\varphi_{0}$ of $\psi_{0}(\varphi )$ with even multiplicity *m*. Expanding $\psi_{0}(\varphi )$ and $\psi_{1}(\varphi )$ around $\varphi_{0}$ yields


$$\begin{array}{cc} \psi (r,\varphi ) & =\psi_{0}(\varphi )+r^{\gamma }\psi_{1}(\varphi )\\ & =\frac{\psi_{0}^{\left(m\right)}(\varphi_{0})}{m!}(\varphi -\varphi_{0}{)}^{m}+O(\varphi -\varphi_{0}{)}^{m+1}+r^{\gamma }\psi_{1}(\varphi_{0})+r^{\gamma }O(\varphi -\varphi_{0}). \end{array}$$

According to the assumptions in (B), we have $\psi_{1}(\varphi_{0})\neq 0$. Now if


$$\begin{array}{c} \psi_{0}^{\left(m\right)}(\varphi_{0})\psi_{1}(\varphi_{0})>0, \end{array}$$

then


$$\begin{array}{c} \psi (r,\varphi )>0\;(\text{or}<0),\;|\varphi -\varphi_{0}|\ll 1. \end{array}$$

Consequently, the equation $\psi (r,\varphi )=\psi_{0}(\varphi )+r^{\gamma }\psi_{1}(\varphi )=0$ admits no real roots within a sufficiently small neighborhood of $\varphi_{0}$.

If


$$\begin{array}{c} \psi_{0}^{\left(m\right)}(\varphi_{0})\psi_{1}(\varphi_{0})<0, \end{array}$$

then the equation ψ(r,φ)=0 possesses two distinct real roots


$$\begin{array}{c} \varphi_{1,2}^{*}=\varphi_{0}\pm {\left(\frac{-m!r^{\gamma }\psi_{1}(\varphi_{0})}{\psi_{0}^{\left(m\right)}(\varphi_{0})}+O(\varphi -\varphi_{0}{)}^{m+1}+r^{\gamma }O(\varphi -\varphi_{0})\right)}^{\frac{1}{m}}. \end{array}$$

Because $\varphi_{1,2}^{*}\neq \varphi_{0}$, and


$$\begin{array}{c} \psi_{0}(\varphi )=\frac{\psi_{0}^{\left(m\right)}(\varphi_{0})}{m!}(\varphi -\varphi_{0}{)}^{m}+O(\varphi -\varphi_{0}{)}^{m+1}, \end{array}$$

then


$$\begin{array}{c} \psi_{0}^{\prime }(\varphi_{1,2}^{*})\neq 0. \end{array}$$

Consequently, for every fixed sufficiently small *r* > 0,


$$\begin{array}{c} \frac{\partial \psi }{\partial \varphi }(r,\varphi_{1,2}^{*})\neq 0. \end{array}$$

Thus, for any fixed sufficiently small *r* > 0, two real solutions $\varphi_{1}^{*}$ and $\varphi_{2}^{*}$ to ψ(r,φ)=0 are simple roots (i.e., of multiplicity one).

We prove instability using Chetaev’s theorem. Accordingly, we will construct a Chetaev’s function *V* and a region Ω that satisfy the conditions required for the theorem’s application (see [21], Chapter 13]).

Specifically, Chetaev’s theorem requires the existence of a nonempty open set Ω containing the origin in its boundary, a continuously differentiable function *V* defined on Ω, and a positive constant *a* such that:

(1) *V* > 0 in Ω and *V* = 0 on $\partial \Omega \cap B_{a}(0)$;
(2) $\dot{V}>0$ in Ω;
(3) 0∈∂Ω.

We let


$$\begin{array}{c} V=\delta r^{2\alpha +\gamma }-r^{2\alpha }\psi^{2}(r,\varphi ), \end{array}$$

where δ>0 is a parameter to be chosen later so that, within a region Ω (to be defined), the following hold: *V* > 0 in Ω, *V* = 0 in ∂Ω and $\dot{V}>0$ in Ω.

Define


$$\begin{array}{c} \begin{array}{ccc} D_{a} & = & \{(r,\varphi ):V\geq 0,r<a\},\\ D_{a}^{+} & = & \{(r,\varphi ):V\geq 0,r<a,\frac{\partial \psi }{\partial \varphi }(r,\varphi )>0\},\\ D_{a}^{-} & = & \{(r,\varphi ):V\geq 0,r<a,\frac{\partial \psi }{\partial \varphi }(r,\varphi )<0\}, \end{array} \end{array}$$

where *a* is a suitably chosen positive constant. Consistent with the hypotheses of (B), there exists a zero $\varphi_{0}$ of $\psi_{0}(\varphi )$ such that


$$\begin{array}{c} \psi_{0}^{\left(m\right)}(\varphi_{0})\psi_{1}(\varphi_{0})<0, \end{array}$$

where *m* > 1 is the multiplicity of the zero $\varphi_{0},$ we have that, for any fixed sufficiently small *r* > 0, the equation ψ(r,φ)=0 has two simple real roots $\varphi_{1}^{*}$ and $\varphi_{2}^{*}$ near $\varphi_{0}$, and we may assume that


$$\begin{array}{c} \frac{\partial \psi }{\partial \varphi }(r,\varphi_{1}^{*})>0,\;\frac{\partial \psi }{\partial \varphi }(r,\varphi_{2}^{*})<0. \end{array}$$ (11)

It follows that $D_{a}\neq \emptyset$, because


$$\begin{array}{c} V(r,\varphi_{1}^{*})=\delta r^{2\alpha +\gamma }-r^{2\alpha }\psi^{2}(r,\varphi_{1}^{*})=\delta r^{2\alpha +\gamma }\geq 0. \end{array}$$

Consequently, (11) implies that both $D_{a}^{+}$ and $D_{a}^{-}$ are nonempty subsets of $D_{a}$.

In $D_{a}$, we have


$$\begin{array}{c} \delta r^{2\alpha +\gamma }-r^{2\alpha }\psi^{2}(r,\varphi )\geq 0, \end{array}$$

i.e.,


$$\begin{array}{c} \psi^{2}(r,\varphi )\leq \delta r^{\gamma }\leq \delta a^{\gamma }<\delta \end{array}$$ (12)

for 0 < *a* < 1. Our next objective is to choose δ such that, for sufficiently small *r* > 0, we have $D_{a}=D_{a}^{+}\cup D_{a}^{-}$ and $\overset{―}{D_{a}^{+}}\cup \overset{―}{D_{a}^{-}}=\{r=0\}$. A sufficient condition for this is to show that


$$\begin{array}{c} \frac{\partial \psi }{\partial \varphi }(r,\varphi )\neq 0 \end{array}$$

in $D_{a}$ for every *r* > 0 sufficiently small. In fact, Assume that for $r=\tilde{r}>0$ sufficiently small, there exists some $\varphi =\tilde{\varphi }$ such that


$$\begin{array}{c} \frac{\partial \psi }{\partial \varphi }(\tilde{r},\tilde{\varphi })=0 \end{array}$$

Since all real roots of ψ(r,φ)=0 are simple, we must have $\psi (\tilde{r},\tilde{\varphi })\neq 0.$ Choosing $0<\delta \leq \psi^{2}(\tilde{r},\tilde{\varphi })$ and applying inequality (12) leads to the contradiction


$$\begin{array}{c} \delta \leq \psi^{2}(\tilde{r},\tilde{\varphi })<\delta . \end{array}$$

Hence, our assumption is false, and we conclude that $D_{a}=D_{a}^{+}\cup D_{a}^{-}.$

If $\frac{\partial \psi }{\partial \varphi }(r,\varphi )\neq 0$ for all *r* > 0 and φ∈ℝ, then δ>0 can be chosen arbitrarily. In this case, we likewise obtain $D_{a}=D_{a}^{+}\cup D_{a}^{-}.$ Moreover, it is evident that $\overset{―}{D_{a}^{+}}\cup \overset{―}{D_{a}^{-}}=\{r=0\}$, so the origin lies in the closure of each component.

We now choose a connected component Ω of $D_{a}^{-}$. Since $\Omega \subset D_{a}$, we have V≥0 in Ω, and on the portion of ∂Ω where *r* > 0, we have either *V* = 0 (on ψ=0) or *r* = *a* (where *V* > 0 if δ is chosen small). More precisely, on the boundary curves ψ=0, we have $V=\delta r^{2\alpha +\gamma }>0$ unless *r* = 0, so *V* is strictly positive on those curves; on *r* = *a*, $V=\delta a^{2\alpha +\gamma }-a^{2\alpha }\psi^{2}(a,\phi )>0$ if $\delta a^{\gamma }>\max_{\varphi }\psi^{2}(a,\varphi )$. Thus *V* > 0 in Ω and *V* = 0 on the intersection of ∂Ω with a sufficiently small neighborhood of the origin (where *r* = 0). Also, 0∈∂Ω by construction.

It remains to verify $\dot{V}>0$ in Ω.

We now compute the time derivative of *V* along solutions of the Hamiltonian system for *H*. Evaluating the Poisson bracket yields


$$\begin{array}{cc} \dot{V} & =\{V,H\}=\{\delta r^{2\alpha +\gamma }-r^{2\alpha }\psi^{2}(r,\varphi ),H\}\\ & =\delta (2\alpha +\gamma )r^{2\alpha +\gamma -1}\{r,H\}-2r^{\alpha }\psi (r,\varphi )\{r^{\alpha }\psi (r,\varphi ),H\}\\ & =-\delta (2\alpha +\gamma )r^{2\alpha +\gamma -1}\frac{\partial H}{\partial \varphi }-2r^{\alpha }\psi (r,\varphi )\{r^{\alpha }\psi (r,\varphi ),O(r^{\alpha +\gamma +\frac{1}{2}})\}\\ & =-\delta (2\alpha +\gamma )r^{2\alpha +\gamma -1}\left(r^{\alpha }\frac{\partial \psi }{\partial \varphi }(r,\varphi )+O(r^{\alpha +\gamma +\frac{1}{2}})\right)\\ & \;\;-2r^{\alpha }\psi (r,\varphi )O(r^{2\alpha +\gamma -\frac{1}{2}})\\ & =-\delta (2\alpha +\gamma )r^{3\alpha +\gamma -1}\frac{\partial \psi }{\partial \varphi }(r,\varphi )+O(r^{3\alpha +\gamma -\frac{1}{2}}), \end{array}$$

here $\dot{V}$ denotes the derivative along solutions. Clearly, $\dot{V}>0$ in $D_{a}^{-}$. Selecting a single connected component Ω of $D_{a}^{-}$, the function *V* satisfies all conditions of Chetaev’s theorem within Ω. It then follows from Chetaev’s theorem that the equilibrium *r* = 0 is unstable. This completes the proof of Theorem 1.

Now we turn to the proof of Theorem 2.

**Proof of Theorem 2** In light of the assumptions in Theorem 2, we may, without loss of generality, take it that


$$\begin{array}{c} \psi_{0}^{\left(m\right)}(\varphi_{0})<0,\;\psi_{1}^{\prime }(\varphi_{0})<0. \end{array}$$ (13)

Expanding $\psi_{0}(\varphi )$ at $\varphi_{0}$ yields that


$$\begin{array}{c} \psi_{0}(\varphi )=\frac{\psi_{0}^{\left(m\right)}(\varphi_{0})}{m!}(\varphi -\varphi_{0}{)}^{m}+O(\varphi -\varphi_{0}{)}^{m+1}, \end{array}$$ (14)

where *m* > 1 is the multiplicity of the zero $\varphi_{0}$ of the function $\psi_{0}(\varphi )$, and *m* is odd.

From (13) and (14), there exists a number $0<\delta_{1}<1$ such that


$$\begin{array}{c} \psi_{0}^{\prime }(\varphi )=\frac{\psi_{0}^{\left(m\right)}(\varphi_{0})}{(m-1)!}(\varphi -\varphi_{0}{)}^{m-1}+O(\varphi -\varphi_{0}{)}^{m}<0 \end{array}$$

holds for any φ satisfying $0<|\varphi -\varphi_{0}|<\delta_{1}$. Since $\psi_{1}^{\prime }(\varphi_{0})<0,$ there exists $\delta_{2}>0$ such that $\psi_{1}^{\prime }(\varphi )<0,$ for any φ satisfying $|\varphi -\varphi_{0}|<\delta_{2}.$

Let $\delta =\min\{\delta_{1},\delta_{2}\}$, and


$$\begin{array}{c} S=\left\{(\varphi ,r):|\varphi -\varphi_{0}|<\delta ,\;r>0\;\text{small}\;\text{enough}\right\}. \end{array}$$

In *S*, we have


$$\begin{array}{cc} \dot{r} & =-H_{\varphi }=-r^{\alpha }\psi_{0}^{\prime }(\varphi )-r^{\alpha +\gamma }\psi_{1}^{\prime }(\varphi )+O(r^{\alpha +\gamma +\frac{1}{2}})\\ & =-\frac{\psi_{0}^{\left(m\right)}(\varphi_{0})}{(m-1)!}(\varphi -\varphi_{0}{)}^{m-1}r^{\alpha }-r^{\alpha +\gamma }\psi_{1}^{\prime }(\varphi )\\ & \;\;+O(r^{\alpha +\gamma +\frac{1}{2}})+O(\varphi -\varphi_{0}{)}^{m}>0, \end{array}$$ (15)

and


$$\begin{array}{cc} \dot{\varphi } & =H_{r}=\alpha r^{\alpha -1}\psi_{0}(\varphi )+O(r^{\alpha -\frac{1}{2}})\\ & =\alpha r^{\alpha -1}\left(\psi_{0}(\varphi )-\psi_{0}(\varphi_{0})\right)+O(r^{\alpha -\frac{1}{2}})\\ & =\alpha r^{\alpha -1}\psi_{0}^{\prime }(\varphi_{1})(\varphi -\varphi_{0})+O(r^{\alpha -\frac{1}{2}}), \end{array}$$ (16)

where $\varphi_{1}$ satisfies $0<|\varphi_{1}-\varphi_{0}|<\delta_{1}.$

Since $\psi_{0}^{\prime }(\varphi_{1})<0,$ it follows directly from (15) and (16), it can be deduced that any solution (φ(t),r(t)) of (8) with (φ(0),r(0))∈S always stays in *S* and $r^{\prime }(t)>0$ for all t≥0. This guarantees the Lyapunov instability of the equilibrium *r* = 0.

### Examples

The main results presented in this work serve to extend and synthesize a series of existing findings in the literature.

### Examples 1

Consider the case of fourth-order resonance investigated in [18], our criteria are directly applicable to this scenario, providing explicit conditions for both stability and instability. In this particular case, the Hamiltonian function simplifies to the following


$$\begin{array}{c} H=(1-\cos4\varphi )r^{2}+\left(s+k(1-\cos4\varphi )\right)r^{3}+O(r^{4}). \end{array}$$

[18] showed that stability of the equilibrium *r* = 0 depends on the sign of *s*, for *s* = 1 it is stable, whereas for s=−1 it is unstable.

However, applying Theorem 1 to this setting, we identify the functions $\psi_{0}(\varphi )$ and $\psi_{1}(\varphi )$ as


$$\begin{array}{c} \psi_{0}(\varphi )=1-\cos4\varphi ,\;\psi_{1}(\varphi )=s+k(1-\cos4\varphi ). \end{array}$$

It is clear that $\psi_{0}(\varphi )$ vanishes at $\varphi_{j}=\frac{j\pi }{2},j\in \mathbb{Z}$, and each zero is of multiplicity two. Hence, for the case *s* = 1, we have


$$\begin{array}{c} \frac{d^{2}\psi_{0}}{d\varphi^{2}}(\varphi_{j})\psi_{1}(\varphi_{j})>0, \end{array}$$

it then follows from (A) of Theorem 1 that the equilibrium is stable. Conversely, if s=−1,


$$\begin{array}{c} \frac{d^{2}\psi_{0}}{d\varphi^{2}}(\varphi_{j})\psi_{1}(\varphi_{j})<0 \end{array}$$

holds, according to the (B) of Theorem 1, the equilibrium is unstable.

### Examples 2

Consider the Hamiltonian


$$\begin{array}{c} H(r,\theta ,t)=\omega r+r^{2}{\left(\cos2\theta -\frac{1}{2}\right)}^{2}+r^{3}\Phi_{1}(\theta ,t)+r^{4}\Phi_{2}(r,\theta ,t), \end{array}$$

where $\psi_{0}(\theta )={\left(\cos2\theta -\frac{1}{2}\right)}^{2}$ has zeros of multiplicity two. This example satisfies the hypotheses of Theorem 1 but does not satisfy the conditions of Cabral and Meyer ([16]) (which requires simple zeros) or Bardin ([15]) (which requires multiplicity one). Therefore, it falls outside the scope of previously published results and illustrates the broader applicability of our stability criteria.

## Conclusion

In this paper, we have established two general stability criteria (Theorems 1 and 2) for equilibrium solutions in degenerate periodic Hamiltonian systems with one degree of freedom. For the class of degenerate systems in which the normalized action-dependent part vanishes identically and the leading angular coefficient possesses only zeros of finite multiplicity, our theorems provide a complete stability classification except for the measure-zero set of parameters where $\psi_{0}$ and $\psi_{1}$ share common zeros. The criteria are applicable to resonances of arbitrary order and provide easily verifiable conditions, thereby eliminating the need for the intricate computations required in earlier analyses.

The main limitations of our results are: (i) the case where $\psi_{0}$ and $\psi_{1}$ share common zeros remains open, and (ii) the analysis is restricted to one degree of freedom. Potential future research directions include: (1) extension to more degrees of freedom; (2) treatment of the common-zero case via blow-up or degenerate KAM theory; (3) numerical verification of the stability criteria on concrete physical models; and (4) application to celestial-mechanics problems with degenerate resonances.
